# Organoid of ovarian cancer: genomic analysis and drug screening

**DOI:** 10.1007/s12094-019-02276-8

**Published:** 2020-01-14

**Authors:** H.-D. Liu, B.-R. Xia, M.-Z. Jin, G. Lou

**Affiliations:** 1 Department of Gynecology, Harbin Medical University Cancer Hospital, Harbin, Heilongjiang 150081 People’s Republic of China; 2 Shanghai Jiao Tong University School of Medicine, Shanghai, 200025 People’s Republic of China

**Keywords:** Organoid, Targeted therapy, Immunotherapy, Drug screening, Ovarian cancer

## Abstract

Ovarian cancer is the most common malignant tumors of the female reproductive system, and its standard treatments are cytoreductive surgery and platinum-based adjuvant chemotherapy. Great advances have been achieved in novel treatment strategies, including targeted therapy and immunotherapy. However, ovarian cancer has the highest mortality rate among gynecological tumors due to therapeutic resistance and the gap between preclinical data and actual clinical efficacy. Organoids are a 3D culture model that markedly affects gene analysis, drug screening, and drug sensitivity determination of tumors, especially when used in targeted therapy and immunotherapy. In addition, organoid can lead to advances in the preclinical research of ovarian cancer due to its convenient cultivation, good genetic stability, and high homology with primary tumors.

## Introduction

Cancer is one of the leading causes of deaths worldwide [1]. Ovarian cancer (OC) has the highest mortality rate among gynecological tumors that threaten women’s health and life [2]. Most cases (70%) are diagnosed at an advanced stage because the clinical manifestations of early OC are hidden or unspecific [3]. Traditional and novel treatment schemes for this illness have progressed in the past decades, but the lack of early diagnosis and the poor efficiency of postoperative chemotherapy restrict the improvement in the 5-year survival rate of patients with OC [3]. Therefore, research on OC focuses on determining highly specific and sensitive tumor markers for early diagnosis and prognosis evaluation (diagnostic aspect) and on exploring new strategies (therapeutic aspect), such as targeted therapy and immunotherapy. Preclinical models that can accurately recapitulate the biological characteristics of tumors in vivo are essential in this process. OC cell lines used to have a dominant role in OC biology, but have been gradually replaced by patient-derived xenograft (PDX). In the era of precision medicine, preclinical research platform derived from each individual has become indispensable, and high-throughput genomic analysis has been widely used to search for effective personalized treatment methods.

Organoid is a powerful tool for precision medicine and drug screening [4, 5]. This technique can maintain the characteristics of tumor and its microenvironment in vivo to the greatest extent and can rectify the shortcomings of single cell lines in testing new drugs. The organoid maintains homology with primary tumors for a long time; hence, its drug sensitivity is better than that of cell lines during drug screening. Tumor-like organs are easy to replicate and pass on to form a biobank, which can be used for large-throughput gene analysis and drug screening. These organs can maintain the genetic heterogeneity of tumors and can mimic a hypoxic microenvironment. In this review, we discuss and compare the advantages and disadvantages of four OC preclinical model categories, summarize the application of organoid for genomic analysis and drug screening in targeted therapy and immunotherapy, and highlight the potential application of organoid in OC preclinical research.

## Preclinical models in OC research

The current preclinical models of OC mainly consist of cell lines, in vivo animal models, spheroids, and organoids, among which, cell lines and in vivo animal models are widely used. Spheroids are seldom used because of their difficult cultivation and the lack of control groups. The organoid is a novel research model that is simple to cultivate, has cancer homology, and has been gradually applied to gene analysis, drug screening, and other types of research. Multiple models are complementary. On the basis of the merits and demerits of different models, the organoid allows preclinical data to accurately reflect OC and shorten the distance between experimental and clinical applications (Table 1).

**Table 1:** Advantages and disadvantages of various preclinical models

	Advantages	Disadvantages
Cancer cell lines	Simple and easy to operate Can be used for high-throughput drug screening	Changes in the genome structure Cannot accurately represent the pathological characteristics of primordial cells
Patient-derived xenograft (PDX)	Better representation of the nature of the tumor Preserves interaction with primary tumors Preserves the heterogeneity of primary tumors	Time consuming, expensive, and low success rate Existence of ethical problems Limitation of immunological activity in recipient mice Difficulty of cultivating low-grade malignant tumors Certain timeliness
Genetically engineered rat model (GEMMS)	Complete immune function Evaluation of mutation sites in different tumors	Complex establishment process Cannot completely simulate a particular disease at the molecular level
Non-mouse cancer model		
Drosophila melanogaster	Physiologically and pathologically similar to mammals Highly similar tumor signal transduction pathways to those in humans	No acquired immune function Short life cycle Cannot reflect cancer changes over time
Nematode	Grows fast and multiplies in large numbers Transparent body and easy to observe Completed genome sequencing	Structure is too simple Low similarity with human structure
Zebrafish	High homology with human genome Tenacious vitality and easy to cultivate Short generation Clear genetic background	Source species are difficult to determine Modeling engineering needs to be improved
Spheroid	Study on drug resistance and metastasis Produces PDX to evaluate the tumorigenicity of primary tumors Enrichment of stem cell-like cells in spheroid	Lack of control group Normal epithelial cells cannot be cultured Low success rate of cultivation
Organoids	Can be cultured on a large scale and high-throughput drug screening Various subtypes can be cultured Tumor gene expression profile can be maintained in a long-term culture Maintains good genetic stability and tumor heterogeneity Better simulation of hypoxic microenvironment of tumors	Lacks matrix, immune cells, and blood vessels Expensive Low success rate of culture of some subtypes of tumors

### Cancer cell lines

Cell lines are special types of organizations with unlimited proliferation potential on plastic Petri dishes. More than 50 OC cell lines have been established and used in preclinical research [6], and the common ones are SKOV-3, A2780, OVCAR-3, CAOV3, and IGROV-1 [7]. Cell lines are simple, easy to operate, usually used to screen antineoplastic drugs and histological subtypes of OC in vitro to study anticancer pathways, and easily employed to conduct large-scale and high-throughput drug screening. For example, cell lines can be used in investigating the effects of genes on cell survival, proliferation, and kinase-specific inhibitors because of their immortality and accessibility [8].

However, these cell lines have been passed on for several generations or even decades in vitro. Owing to highly evolutionary selection, the genomic structure of cell lines inevitably changes. Stromal cells and extracellular matrix proteins of tumor tissues are lost during in vitro passage, and only the cells that have the same phenotype and successfully adapted to the environment can survive, resulting in the loss of tumor heterogeneity [9]. As a result, these cell lines have different gene expression profiles compared with primary tumors and thus cannot accurately represent the genotype and pathological characteristics of the latter. The advantages of OC cell lines have rendered their involvement in OC research. However, the reliability of cell line source data has been questioned due to unsatisfactory results from clinical trials. Primary cells are still the choice of many researchers.

### Animal model in vivo

The commonly used in vivo animal models include the cell line allotransplantation model and PDX. The former transplants cell lines into immunodeficient mice and has been widely used in OC drug screening. Cell transplantation methods include subcutaneous, in situ, and intraperitoneal inoculations. Considering that precision medicine emphasizes the importance of patient-derived models, researchers have upgraded cell line xenotransplantation models and called them PDX. Fresh tumor cells or tissues of resected patients were implanted into the immunodeficient mice, PDX, to preserve genome integrity and tumor heterogeneity [10, 11]. PDX transplants the patient’s tumor fragments into immunodeficient mice and can be used for resected tissue, biopsy, ascites/pleural effusion, and pleural sampling. Subcutaneous inoculation is the main method of inoculation. PDX has been established from various types of cancers [12] and has advantages, such as retention of the interaction between tumors and tissues and good representation of the nature of tumors. In the established PDXs of melanoma, breast cancer, and pancreatic cancer, the chemotherapy response is similar to that from the patient’s source and could accurately reflect the natural state of the tumor [13, 14]. Cytogenetic analysis revealed that compared with cell lines, PDX retains the chromosome structure of tumor tissues [15]. However, PDX use is limited in the study of ovarian cancer [16, 17]. At present, this model is only applied in the early stage of model establishment and chemotherapy response observation. Colombo’s team [18] established 35 large-scale ovarian cancer PDXs. The pathological types include clear cell carcinoma, mucinous carcinoma, and carcinosarcoma. Ricci’s team [19] established some special PDXs of ovarian cancer, such as recurrent tumor and Akt and ERK pathway platinum-resistant tumor, which verified the efficacy of antitumor multi-drug (paclitaxel, bevacizumab, and MEK inhibitor). The results show that the combination of three drugs provides better tolerance and antitumor effect than the combination of two drugs. In addition, PDX is an important tool for the development of new second-line antitumor drugs. Another team established a series of ovarian cancer PDX that banks with different cisplatin sensitivity. The results confirmed that the PDX of ovarian cancer provides a platform for DNA damage repair test and is an important tool in evaluating the response of patients to cisplatin treatment [20].

Ovarian cancer PDX shows a good application prospect and is beneficial for the analysis of ovarian cancer heterogeneity, drug screening, individualized treatment, and recurrence analysis. Furthermore, PDX helps improve clinical efficacy, high recurrence, and early diagnosis rate. However, compared with the simple monolayer cultures of cell lines, PDX is time consuming, expensive, and has a relatively low success rate. Moreover, the effect of immunity on tumors is ignored, and the evaluation of pharmacodynamics and toxicity is limited because only immunodeficient mice are used. Thus, improving PDX to study the immune mechanism of tumors is necessary. Another disadvantage is that cell lines and PDX share the same characteristics. Both easily cultivate tumors with high malignancy, but have difficulty in cultivating tumors with low malignancy [21–23]. These disadvantages have hindered the wide application of PDX.

### Spheroid

Relative to the 2D structure of cell lines, the globule is a globular cell aggregate that remains as a floating 3D structure. Compared with cell lines and PDX, spheroids can greatly restore the microenvironment of tumors and maintain similarity to the original tumors [24–26]. Ovarian cancer development includes the separation of cancer cells from the fallopian tube or carcinoma in situ, their proliferation to the peritoneal cavity in the form of single cells or globules, and their attachment to the epidermis of the greater omentum, large intestine, and abdomen [27, 28]. Researchers began to explore the spheroids derived from cancer ascites. Malignant ascites, which contain tumor cells suspended into a sphere and are rich in ovarian cancer stem cells [29, 30], are a challenge for ovarian cancer treatment. Functional enrichment of stem cell-like cell populations can be achieved through globular cultures. The spheroid culture is used in studying drug resistance and metastasis of cancer due to the cancer stem cell theory, which holds that the malignant phenotype of cancer is mainly mediated by the stem cell-like part [31–33]. Detailed analysis of ovarian cancer spheroids revealed the mutual regulatory pathway of ALDH1 and Sox2 involved in ovarian cancer stem cells [34]. Another study group tested the sensitivity of cisplatin, ALDH inhibitor, and JAK2 inhibitor by detecting the ALDH1 and CD133 of globular stem cells. The results showed that globular stem cells sustain their resistance to cisplatin/ALDH inhibitor, although the expression of ALDH is low, and that of CD133 is completely lost. However, spheres resistant to cisplatin/JAK2 inhibitors have enriched ALDH+91 cells [35]. In the above-mentioned studies, the culture of spheroids originated from the ascites in the late stage of cancer. However, the globule cannot cultivate the tissues of normal fallopian tube (FT) and ovarian epithelium (OSE), resulting in a lack of control group for comparison. Moreover, the destruction of cell–cell adhesion can easily induce the dysfunctional apoptosis of epithelial cells and consequently reduce the success rate of OC globular model culture [7].

### Organoid

The organoid is a 3D tissue model directly induced by stem cells and a newly emerged preclinical model. Its application has been extended to many fields. At present, the organoid can be produced from primary prostate, colon, and pancreatic cancers [36–38] (Table 2). The OC organoid can multiply normal and precancerous cells, and its success rate is higher than those of PDX and spheroid [7].

**Table 2:** Organoids from different tissues of 2019

Tissue	Cancer type	Source of organoid	References
Lung	Non-small cell lung cancer (NSCLC)	Human primary tumor and patient-derived xenograft (PDX)	Tsao ([39], p 11)
	Lung cancer	Human primary tumor	Ishii ([40], p 8)
Pancreas	Pancreatic cancer	Human primary tumor	Knudsen ([41], p 11) Saif ([42], p 7) Haibe-Kains ([43], p 1)
		Human primary tumor and normal pancreas	Tuveson ([44], p 11)
		Splenic xenograft mouse	Nakamura ([45], p 5)
	Pancreatic ductal adenocarcinoma (PDAC)	PDX	Kim ([46], p 9)
		Human primary tumor	Hippo ([47], p 6) Welsch ([48], p 6)
Esophagus	Esophageal adenocarcinoma	Barrett's esophagus (BE) tissue of mice	Quante ([49], p 8)
Stomach	Gastric cancer	Malignant ascites of gastric cancer	Zhan ([50], p 11)
		Human primary tumor	Yu ([51], p 5)
		Human primary tumor and normal stomach	Zavros ([52], p 1)
Breast	Breast cancer	Human primary tumor	Raouf ([53], p 9) Corsi ([54], p 7) Skala ([55], p 6)
		Human primary tumor and decellularized rat	Bruno ([56], p 9)
	The heterogeneity of triple-negative breast cancer (TNBC)	Human primary tumor	Park ([57], p 9)
Brain	Head and Neck Cancer	Normal and tumor patient-material	Oliveira ([58], p 11)
	Head and neck squamous cell carcinoma (HNSCC)	Human primary tumor	Clevers ([59], p 7)
	glioblastoma (GBM)	Patient-derived glioma stem cells (GSCs) and human embryonic stem cell (hESC)	Fine ([60], p 5)
Prostate	Prostatic cancer	Human primary tumor and metastatic cell lines	Kotula ([61], p 9)
		Mouse prostate	Sawyers ([62], p 7)
		Genetically engineered mouse models (GEMMs)	Goodrich ([63], p 6)
Bladder	Bladder cancer	Urine samples of dogs	Sasaki ([64], p 9)
		Human primary tumor and normal mouse urothelium	Clevers ([65], p 3)
Liver	Liver cancer	Human primary hepatic stellate cells (HSCs)	Jung ([66], p 10)
		Directly reprogrammed human hepatocytes (hiHeps)	Hui ([67], p 8)
		Normal human cholang iocyte	Clevers ([68], p 6)
		human primary tumor	Selaru ([69], p 1)
	Cholangiocarcinoma (CCA)	Liver of mouse	Saborowski ([70], p 3)
	Hepatocellular carcinoma (HCC)	Human primary tumor	Ma ([71], p 8)
Colorectal	Colon cancer	Human primary tumor	Muñoz ([72], p 11)
		Human primary tumor and normal colon, normal mouse small intestine and colon	Nold ([73], p 10)
		Human primary tumor and normal colon	Barbáchano ([74], p 7)
	Colorectal cancer	Human primary tumor	Kurisawa ([75], p 10) Kops ([76], p 5) Wiener ([77], p 6) Yao ([78], p 4)
		Mouse intestinal tumor and PDX	Oshima ([79], p 7)
		Human normal colon	Kitagawa ([80], p 6)
	Colon signet-ring cell carcinoma (SRCC)	Human primary tumor	Peng ([81], p 11)
	Traditional serrated adenomas (TSAs)	Human normal colon	Sato ([82], p 10)
	Metastatic colorectal cancer (mCRC)	Human primary tumor	Banerjee ([83], p 2)
Lymph	Non-Hodgkin lymphoma	Human primary tumor	Pérez-Galán ([84], p 7)
Cervix	Cervical clear cell carcinoma (cCCC)	Human primary tumor	Hippo ([85], p 9)
Endometrium	Endometrial cancer	Human primary tumor	Hippo ([86], p 5)
	Endometrial disorders	The primary tissue	Vankelecom ([87], p 8)
Ovary	Ovarian cancer	Human primary tumor and normal tissue	Clevers, Hans ([88], p 5)
Kidney	Renal cancer	Human primary tumor	Bonci ([89], p 2)

**Fig. 1:**
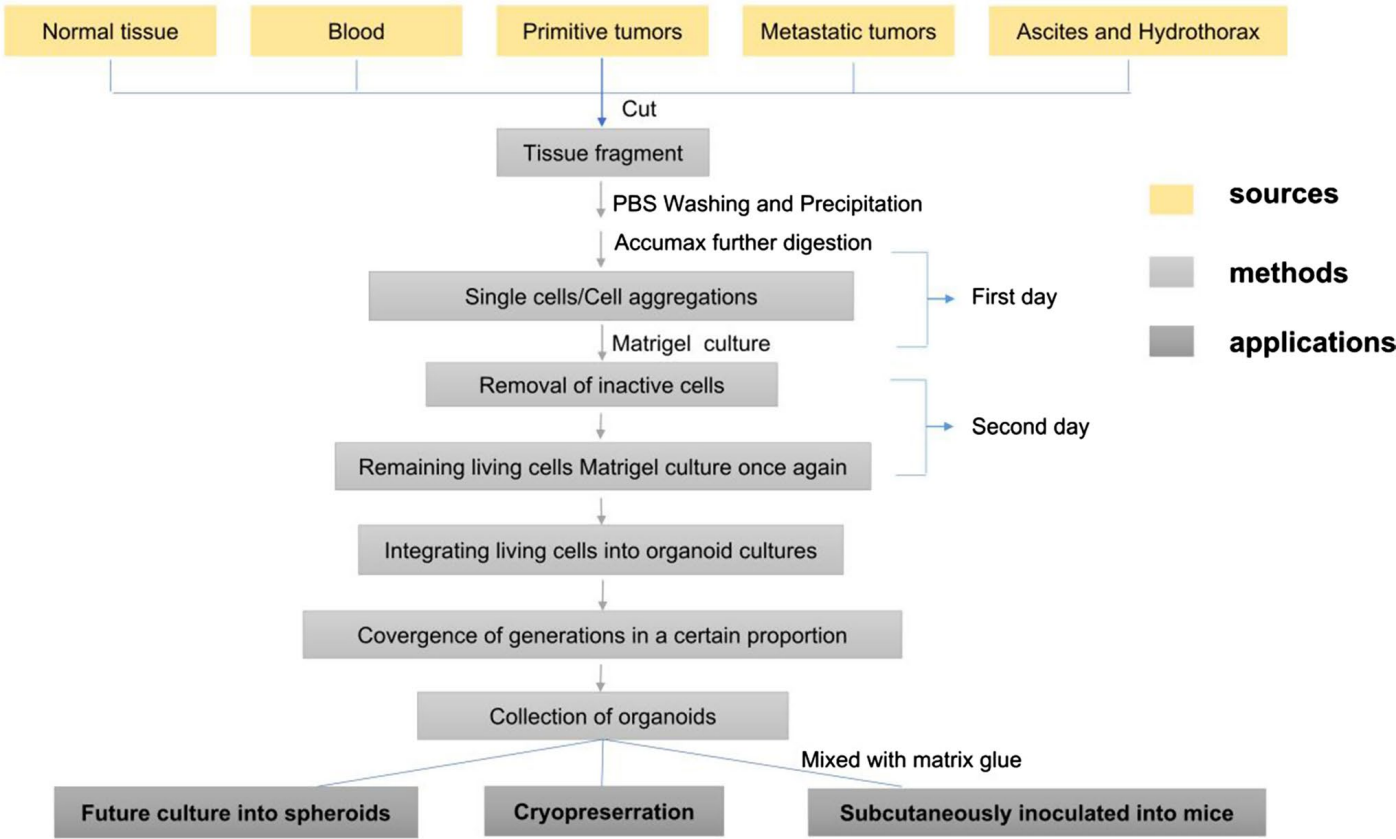
Simple organoid culture

Organoids can be cultured from different tissues of different patients, such as primary and metastatic tumors, blood tissue, ascites, pleural effusion drainage, and normal FT and OSE [90]. Hans Clevers’ team collected 56 organoids from 32 different patients, covering almost all epithelial OC subtypes [90]. Patients with OC are often diagnosed in their advanced stage and sometimes have metastatic lesions. Therefore, multiple organoids can be established in different tissues of patients, proving that this model has good response to the heterogeneity of primary tumors. The team used two OC media for organoid derivation: one with Wnt-conditioned medium (OCwnt medium), and the other without “OCwnt medium.” After two to three generations, a suitable medium for OC organoid culture will appear and thus can be selected. This method improves the success rate of organic matter culture. Moreover, the absolute number of cells recovered from the 3D culture can be increased remarkably by modifying the Matrigel bilayer organoid culture (MBOC) [91, 92] to cope with the digestive resistance and aggregation tendency of OC, and the success rate of organ-like culture can be increased remarkably from 45 to 90% [93] (Fig. 1). The cultured OC-like organs can be cryopreserved, cultured into spheres, or mixed with matrix gel to subcutaneous or orthotopic transplantation to produce PDX for drug sensitivity study in vivo.

## Application of organoid in OC (Fig. 2)

**Fig. 2:**
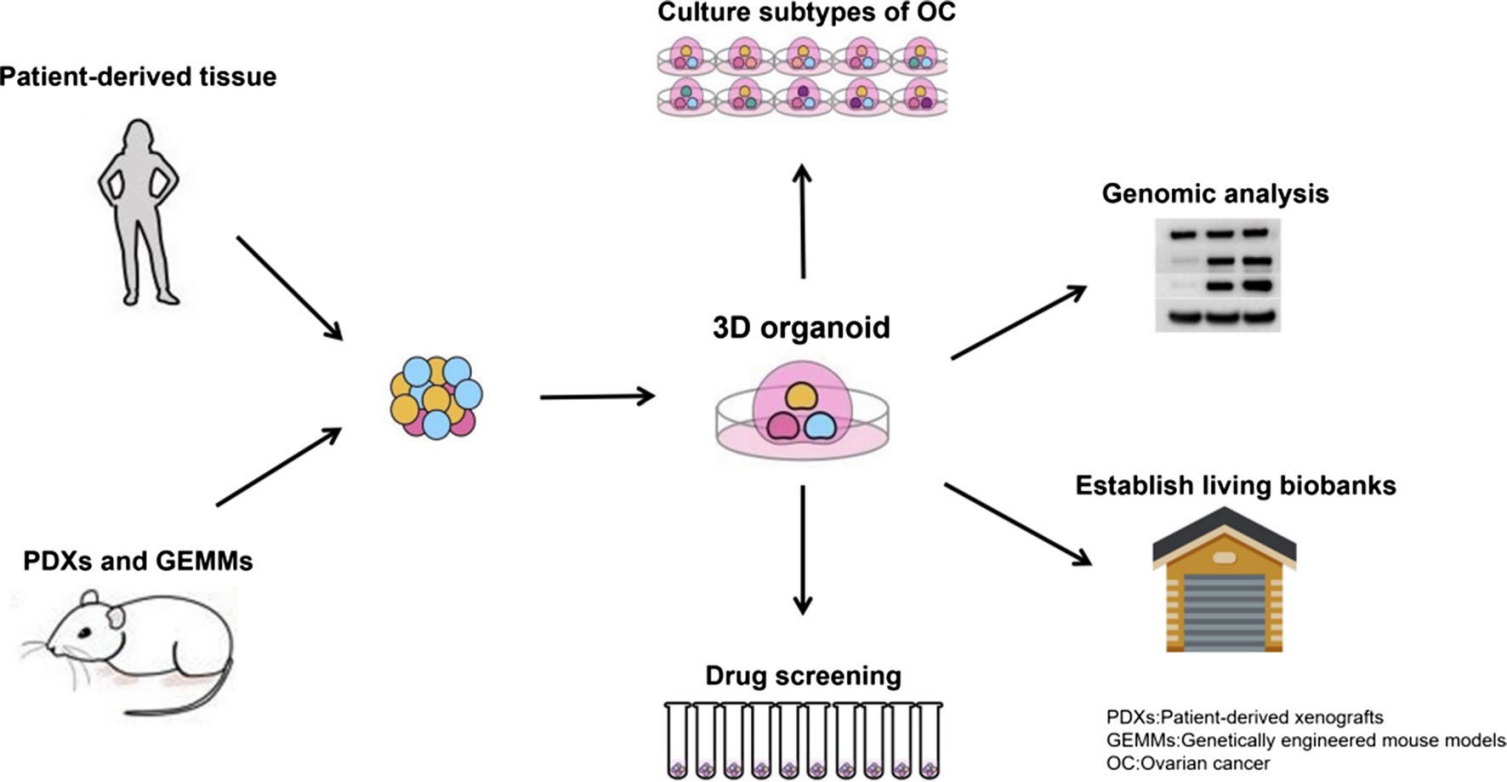
The Sketch of organoid application

### Genomic analysis

The occurrence and development of OC are related to many genes. Studying the changes in OC gene expression profiles through gene analysis is helpful in uncovering disease etiology and finding treatment options. Gene analysis points the way for OC early diagnosis, drug resistance research, and gene therapy. As a representative of primitive tumors, the organoid could be an important tool for gene analysis.

#### Application of organoids in finding tumor-related genes

Long-term organoid culture has good gene stability and can maintain the initial gene expression profile for a long time. Increasing evidence shows that organoids retain somatic cell mutation and copy-number variation (CNV) of original tumors [94, 95]. Hans Clevers’ team validated the high similarity between organoid-like bodies and primitive tumors by analyzing a series of gene levels, such as CNVs, single nucleotide variants (SNVs), and structural variants (SVs) [90]. The results showed that organoids can better reflect the sensitivity of tumors compared with drug treatment regimens. Genome-wide OC analysis using organ-like body platform revealed important genes related to OC development, and this finding will be of great help to our research on gene-directed targeted therapy.

#### The organoid reflects the heterogeneity of tumors better

Ovarian cancer (OC) is a heterogeneous disease, and accurate evaluation of tumor heterogeneity is important in predicting drug resistance and investigating effective treatments. The OC organoid can cultivate various subtypes of OC and reflect the heterogeneity of OC. Hans Clevers’ team performed CNV analysis of organoid in primary and metastatic lesions [90] and showed that genomic changes occur at different time points during tumor evolution. This finding proves that the OC organoid can accurately reflect the heterogeneity of tumors. A new single-cell DNA sequencing method is also used to detect tumors. The heterogeneity between organoid and primary tumors reveals their homology and similarity. This finding confirms that the organoid can capture heterogeneity between tumors and can be used as an effective tool in studying the heterogeneity of OC tumors. Genome and transcriptome analyses of the OC organoid at the single-cell level will allow us to decipher the cellular and molecular bases of heterogeneity in tumors. Responding and studying the heterogeneity of OC through the organoid will affect the development of cancer precision medicine and individualized treatment.

#### Cultivation of organoids in various OC subtypes

Hans Clevers cultured common high-grade serous (HGS) and low-grade malignant OC subtypes, such as low-grade serous (LGS) and clear cell carcinomas (CCC) [90], which filled the defects of traditional preclinical models in low-grade malignant tumors. This finding indicated that organoids can be used in screening and establishing different OC subtypes. The prognosis of patients’ tumors can be quickly classified if the tumor organoids are cultured individually and their histomorphology and gene expression are detected. The organoids generated by these different subtypes can also be stored as a biobank for high-throughput drug screening [94, 96, 97]. At present, several cancer organoid biobanks have been established to identify and test new drugs [36, 94, 98]. Organoids can also cultivate normal FT and OSE tissues, thus solving the problem of no experimental control group caused by spheroids. The OC organoid can be used to produce PDX to evaluate the tumorigenicity of tumors. Previous studies, in situ, subcutaneously transplanted the OC organoid into immunodeficient mice and verified the drug sensitivity of the OC organoid in vivo [90]. Organ-like globules can also be used to determine the drug sensitivity of gynecological tumors [35, 99, 100].

### Drug screening

#### Application in targeted drugs

The tumor organoid can be cultured simultaneously and on a large scale for high-throughput drug screening. Therefore, we can apply the OC organoid in target-directed cancer therapy. Organoids are currently used in the targeted drug therapy of various tumors, and those produced from the liver can be employed as a model in testing the candidate drugs against hepatotoxicity [101–104]. Different responses of androgen receptor positive and negative organoids to enzalutamide were obtained with the use of prostate cancer organoids [36]. Researchers have begun to apply organoids in ovarian cancer. Hans Clevers’ team confirmed that homologous recombinant (HR) defective cells are sensitive to PARP inhibitors, which are also present in the OC organoid [18]. Another team developed the HGS organoid with high success rate for functional analysis of DNA repair and accurate prediction of patients’ clinical response to DNA repair inhibitors [105]. By testing the HR and cross-protection defects of 33 HGS-like organoids in 22 patients, the team confirmed that the functional defects of HR in the organoid are related to the sensitivity of PARP inhibitors regardless of the mutation status of DNA repair genes. In addition, the functional defects of cross-protection of replication are related to the sensitivity of carboplatin, CHK1, and ATR inhibitors. These findings indicate that genome analysis and organ-like function testing can identify targeted DNA damage and repair defects. The OC organoid can be used for DNA repair analysis and therapeutic sensitivity testing, which can immediately evaluate targeted defects in maternal tumors and provide appropriate treatment options.

TP53 gene is another well-known OC target, and its mutation is the main molecular genetic feature in HGS development. One team found that the variant allele frequencies (VAF) for detecting nonsense mutations in TP53 are 70% and 98% in primary tumors and organoid bodies, respectively, whereas the VAF in NF1 increased from 13 to 95% [93] in primary tumors and organoid bodies. The high VAF in organoid bodies indicates that the cancer cells in organoid bodies almost always pass through. Having experienced loss of heterozygosity (LOH), the tumors are composed of cancer cells that are ineffective in TP53. Researchers at Jonsson Comprehensive Cancer Center designed a cell-permeable polypeptide, ReACp53, based on the frequent mutation of TP53 in women with HGS. The ReACp53 can inhibit the formation of amyloid structure of TP53 and restore its function in some cancer cell lines and HGS organoid. Restored TP53 is similar to wild-type TP53 in regulating target gene expression, inhibiting cell proliferation, and promoting cell apoptosis [106]. Furthermore, restored TP53 provides a new idea for future research. Disease models for research were then constructed through organoid-like gene analysis, editing, and modification technologies, such as knockout or overexpression of targeted genes, to validate, search, and develop new gene-targeting drug therapies.

The organoid maintains the gene expression profiles of primitive tumors, can target genes, guide further research on targeted therapy, and be cultured on a large scale to facilitate high-throughput drug screening. At present, almost all kinds of organoid subtypes used to screen targeted drugs for OC subtypes can be cultured to find the best targeted therapy for each subtype.

#### Application of immunotherapy

The immune system is responsible for OC pathogenesis and progression and is effective in destroying and eliminating cancer cells. When OC recurs, drug-resistant cancer cells become highly aggressive, especially those that can protect themselves by inducing immune tolerance [107, 108]. Immunotherapy can be divided into active, passive, and combined immunotherapies that aim to break down the state of immunosuppression and immune tolerance by strengthening the immune recognition and immune-mediated tumor killing of the body. Immune checkpoint inhibitors targeting the PD1/PDL1 axis have been used in clinics. A total of 813 OC clinical trials are ongoing. Among these trials, 72 focus on immune checkpoints, and 27 and 32 are based on anti-PD-1 and anti-PD-L1 antibodies, respectively. Immune checkpoint inhibitors can be used in either single or combination therapy [109]. At present, only few studies have been conducted on tumor immune interaction using tumor organoids [110–112]. Organoids alone cannot generalize the immune system, but researchers have started co-culturing organoids and lymphocytes. Nozaki’s team co-cultured several immune cells (such as inflammatory cells) with organoids and added specific interleukin, and the product can maintain the function of immune cells for a period of time [113]. Zum Walde’s team co-cultured mammary organoids with lymphocytes and proved that these cell cultures can kill three negative breast cancer cells [114]. Another group predicted the efficacy of immunotherapy in individual patients by using the preclinical model of co-culture of mouse-derived gastric cancer organoids and immune cells [115]. These studies have paved the way for the new application of organoid technology in immunotherapy and brought inspiration to ovarian cancer researchers. However, the feasibility of immunotherapy for ovarian cancer by co-culturing lymphocytes and organoids of ovarian cancer is still unknown.

Another form of immunotherapy used in OC therapy is cell adoptive therapy (ACT), an immune technology that uses autologous or allogenic antitumor lymphocytes to induce cancer regression. This therapy has benefited many patients with cancer and aims to express specific T cell receptors or chimeric antigen receptors (CAR-T). CAR-T has achieved revolutionary success in hematological malignancies, but its therapeutic effect on solid tumors is not ideal. This poor effect may be partly due to the lack of understanding of the role of CAR-T cells in the microenvironment of solid tumors. Hypoxia plays a key role in cancer progression and immune editing. The escape of solid tumors from the epidemic surveillance may also be the cause of increased cytotoxicity mediated by CAR-T cells [116, 117]. A study group used EGFRvIII organoids that express CAR-NK-92 cells to analyze the cytotoxicity of tumor antigen specificity and evaluate the efficacy of CAR and tumor specificity. The results showed that NK-92 cells modified by CAR can directly target the ubiquitous epithelial antigen (EpCAM), which can be effectively targeted in multiple organoids [118]. Another group explored the drug sensitivity and cytotoxicity of CAR-T cells by establishing different oxygen gradients in the 3D model of ovarian cancer [119]. The results showed that the OC 3D model can study CAR-T cytotoxicity better than the 2D model. Although the application of organoids in the experiment has not been verified, organoids are the same as other 3D structures. These cell cultures lack blood supply, are prone to anoxia, and are close to or simulate the tumor microenvironment under solid tumor growth [116, 117]. From this point of view, ovarian organoids can simulate the tumor microenvironment and thus are suitable for the study of immunotherapy sensitivity and immunology. However, their use in CAR-T therapy or other immunotherapies is still unverified.

## Prospect and conclusions

The OC organoid is a faithful tumor model that can be used for gene analysis, drug sensitivity prediction, and specific biomarker search. This cell culture can derive OC subtypes, extend them in vitro for a long time, and be used for gene manipulation and drug screening. The organoid is a useful tool in studying gene targeting therapy and provides a good environment for immunotherapy study. These advantages can greatly help in exploring the occurrence and development of OC.

The organoid can establish various OC subtypes, including precancerous cells and normal tissues and therefore can be used to study tumor evolution. Salama’s team used organoids to show the process of *Helicobacter pylori* colonization in gastric epithelium that may cause cell transformation [120]. Scanu’s team used gallbladder organoids to assess the role of *Salmonella* in the development of gallbladder cancer and showed that this infection can activate Akt and MAPK signaling pathways [121]. In the future, the organoid must be employed to study the evolution from normal tissues, precancerous lesions, low-grade malignancy, and finally to high-grade malignancy. The main gene changes are analyzed to achieve early detection, prevention, and treatment. The results will be of great importance for the early screening of OC.

The organoid is suitable for targeted drug research and can be used in combination therapy in the future. Drugs with similar or identical targets have been applied to different organoid subtypes to study their interaction mechanism and identify the combination with the best efficiency and least side effects. A suitable combination of drugs must be determined, and a new combination of targeted therapies must be provided.

We can increase the complexity of organoids by integrating the tumor microenvironment (matrix, vascular system, and immune cells) to provide a good platform for immunotherapy research. Workman’s team developed intestinal organoids containing the nervous system [122]. Ohlund’s team realized the co-culture of organoids and stromal cells [123]. T lymphocytes, not B lymphocytes, are closely related to tumor cloning [124]. T cells donated from healthy individuals can be used to treat patients and kill cancer cells in organoids [113, 114], indicating that these cells can be expanded and activated by organoids. In addition, T lymphocytes can be used to test the therapeutic possibility of introducing healthy blood donor T cells to the tumor cells of patients in vitro. This process could improve the sensitivity of immunotherapy, which is another challenge in the future.

As a new preclinical model, the organoid needs improvements, specifically its success rate. Its tumor microenvironment is single and lacks matrix, blood vessels, and immune cells. Further modifications can render the OC organoid an efficient and robust method for the primary organ culture of gynecological tumors and a highly reliable research approach for targeted therapy and immunotherapy.
